# Pregnancy outcomes of rare autosomal trisomies results in non‐invasive prenatal screening: clinical follow‐up data from a single tertiary centre

**DOI:** 10.1111/jcmm.17245

**Published:** 2022-02-16

**Authors:** Ying Lin, Ping Hu, Hang Li, Chunyu Luo, Dong Liang, Zhengfeng Xu

**Affiliations:** ^1^ State Key Laboratory of Reproductive Medicine Department of Prenatal Diagnosis Nanjing Maternity and Child Health Care Hospital Women’s Hospital of Nanjing Medical University Nanjing Jiangsu Province China

**Keywords:** non‐invasive prenatal screening, pregnancy outcomes, rare autosomal trisomies

## Abstract

This study was performed to assess the association between detection of rare autosomal trisomies (RATs) by non‐invasive prenatal screening (NIPS) and adverse pregnancy outcomes. We retrospectively analyzed women with high‐risk RATs results from January 2014 to December 2020. The women's clinical information was collected, and their pregnancy outcomes were compared with those of women with low‐risk results. In total, 151 (0.24%) RATs results were reported among 62,752 NIPS examinations. Sixty‐five women chose to undergo amniocentesis for confirmation, which revealed 3 cases of true fetal mosaicism for RATs and a positive predictive value of 4.6% (3/65). Among the 139 women with available outcomes, 26 (18.7%) had a preterm birth, 10 (7.2%) underwent pregnancy termination because of fetal defects and 5 (3.6%) had miscarriages. Interestingly, compared with the control group, pregnancies in which NIPS revealed trisomy 16 (T16), T22, T9 and T2 were at higher risk of adverse outcomes, including preterm birth, miscarriage and ultrasound abnormalities. However, the risk of adverse outcomes was comparable between the control group and pregnancies with positive results of T7, T3, T8 and T20. In summary, the risk of adverse pregnancy outcomes was higher in women with specific RATs‐positive NIPS results. Pregnancies with T16, T22, T9 and T2 results, even if false‐positive, should be considered high‐risk pregnancies.

## 1 INTRODUCTION

During the last decade, non‐invasive prenatal screening (NIPS) has become a broadly applied strategy for common aneuploidies.^1^, ^2^ Because massively parallel sequencing‐based NIPS sequences cell‐free DNA from all chromosomes in maternal plasma, it is theoretically able to detect aneuploidies of all chromosomes, including rare autosomal trisomies (RATs). However, whether NIPS should extend to the detection of RATs in clinical practice remains controversial.^3^, ^4^, ^5^, ^6^, ^7^ One important reason for this controversy is that the sensitivity, specificity, false‐positive rate and pregnancy outcome information of the RATs results obtained by NIPS are still limited. Therefore, the clinical benefits of these results remain unclear.

Several previous researchers have reported the RATs results obtained by NIPS, and their data showed that most of these results were false‐positive because of confined placental mosaicism.^3^, ^5^, ^8^ In addition, different conclusions have been drawn regarding the clinical significance of RATs results. In some studies, RATs were associated with fetal growth abnormalities as well as higher miscarriage rates.^3^, ^5^ However, data from other studies showed that RATs were mostly associated with normal pregnancy outcomes.^8^ Although the data regarding RATs are still limited, it has become necessary to clarify the clinical significance of RATs detected by NIPS because of the broadening acceptance of NIPS in clinical practice.

In this study, we retrospectively analyzed 151 RATs results from 62,752 NIPS examinations performed in our centre from January 2014 to December 2020. We reviewed the NIPS results as well as the follow‐up records and compared the pregnancy outcomes with 10,397 low‐risk NIPS results to evaluate the clinical significance of RATs results and to determine whether these results are associated with adverse pregnancy outcomes.

## 2 MATERIALS AND METHODS

### 2.1 Recruitment criteria

This study involved pregnant women who underwent NIPS testing for genome‐wide cell‐free DNA screening in the Medical Genetics Center of the Affiliated Obstetrics and Gynecology Hospital with Nanjing Medical University from January 2014 to December 2020. The inclusion criteria for this study were a pregnancy with a high‐risk RATs report and complete clinical information, and the exclusion criterion was loss to follow‐up. This study was approved by the institutional review board of the Nanjing Maternity and Child Health Care Hospital (approval number NFLZ2019‐KY‐004).

### 2.2 NIPS examinations

Five millilitres of maternal peripheral blood was collected from each participant using EDTA anticoagulant tubes and centrifuged within 8 hours to extract the plasma. The details of the procedure were similar to those described in our previous report.^2^, ^9^ A library was constructed using the BGI protocol and sequenced using the BGISEQ‐500 platform (BGI Group). The cut‐off fetal fraction was set at 3.5%. Fetal chromosomal trisomies for chromosomes 13, 18, 21, X and Y as well as other genome‐wide RATs and subchromosome copy number variants were analyzed.

### 2.3 Outcome collection

**FIGURE 1:**
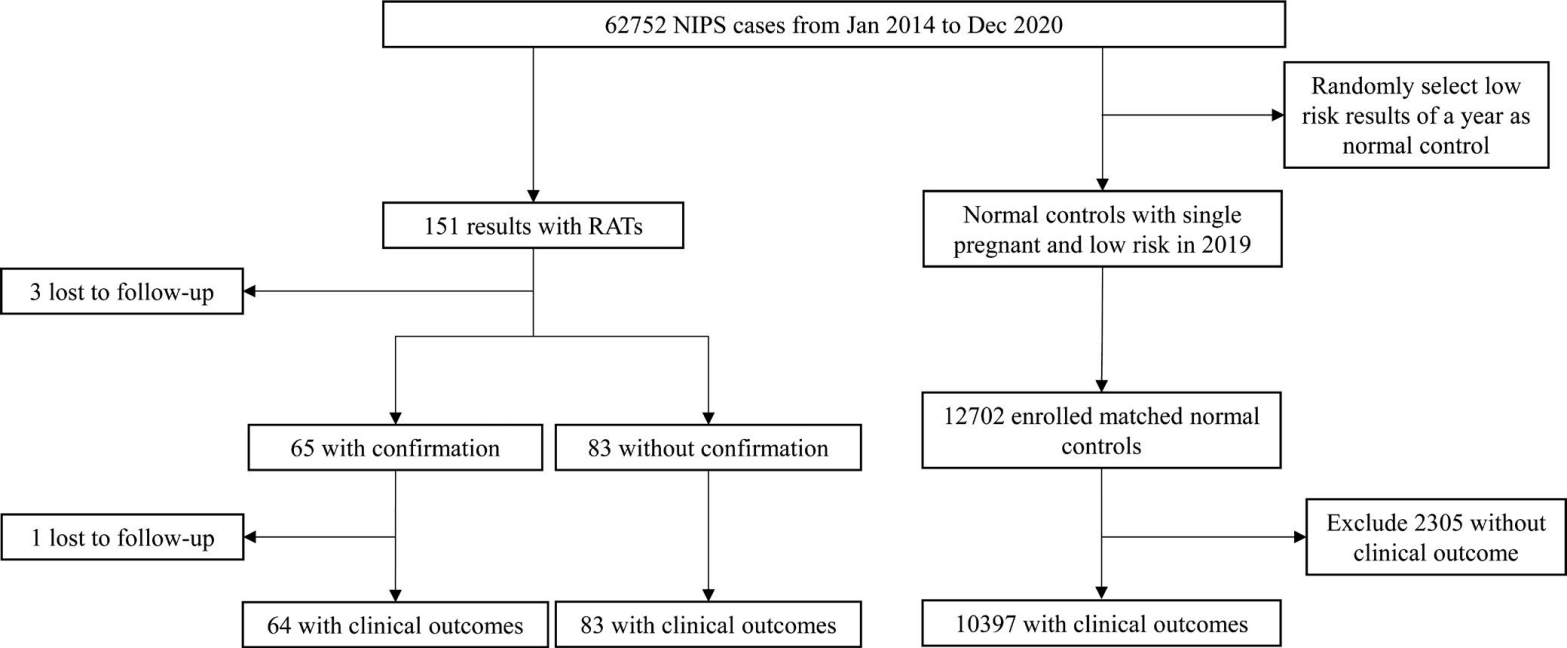
Flow of study design. NIPS, non‐invasive prenatal screening; RATs, rare autosomal trisomies

We retrospectively analyzed the data of women with high‐risk RATs results. The following data regarding these women's maternal and pregnancy characteristics were collected from the laboratory database and clinical records: maternal age, gestational age (GA) at the time of NIPS, NIPS results and pregnancy outcomes (cytogenetic results, ultrasound examination results, miscarriage, termination of pregnancy, GA at the time of delivery and newborn physical examination results). Birthweight percentile was calculated according to NICHD (national institute of child health and human development) (Asian) charts.^10^ Preterm was defined as delivery at <37 weeks of gestation. The pregnancy outcomes for women with low‐risk NIPS results in 2019 were used as comparative controls. Women who had pregnancies with RATs detected by NIPS were recommended to undergo confirmatory invasive prenatal diagnosis using amniocentesis followed by chromosomal microarray analysis. A detailed flow diagram of the study design is shown in Figure 1.

### 2.4 Statistical analysis

Continuous variables such as maternal age and GA are expressed as mean ± standard deviation. The incidence of RATs and pregnancy outcome results are expressed as number and percentage. Differences in outcomes between the RATs group and the low‐risk NIPS group were compared using the *χ* ^2^ test or Fisher's exact test. A *p* value of <0.05 was considered statistically significant. Statistical analysis was performed using SPSS 19.0 (IBM Corp).

## 3 RESULTS

### 3.1 Detection of RATs by NIPS

**FIGURE 2:**
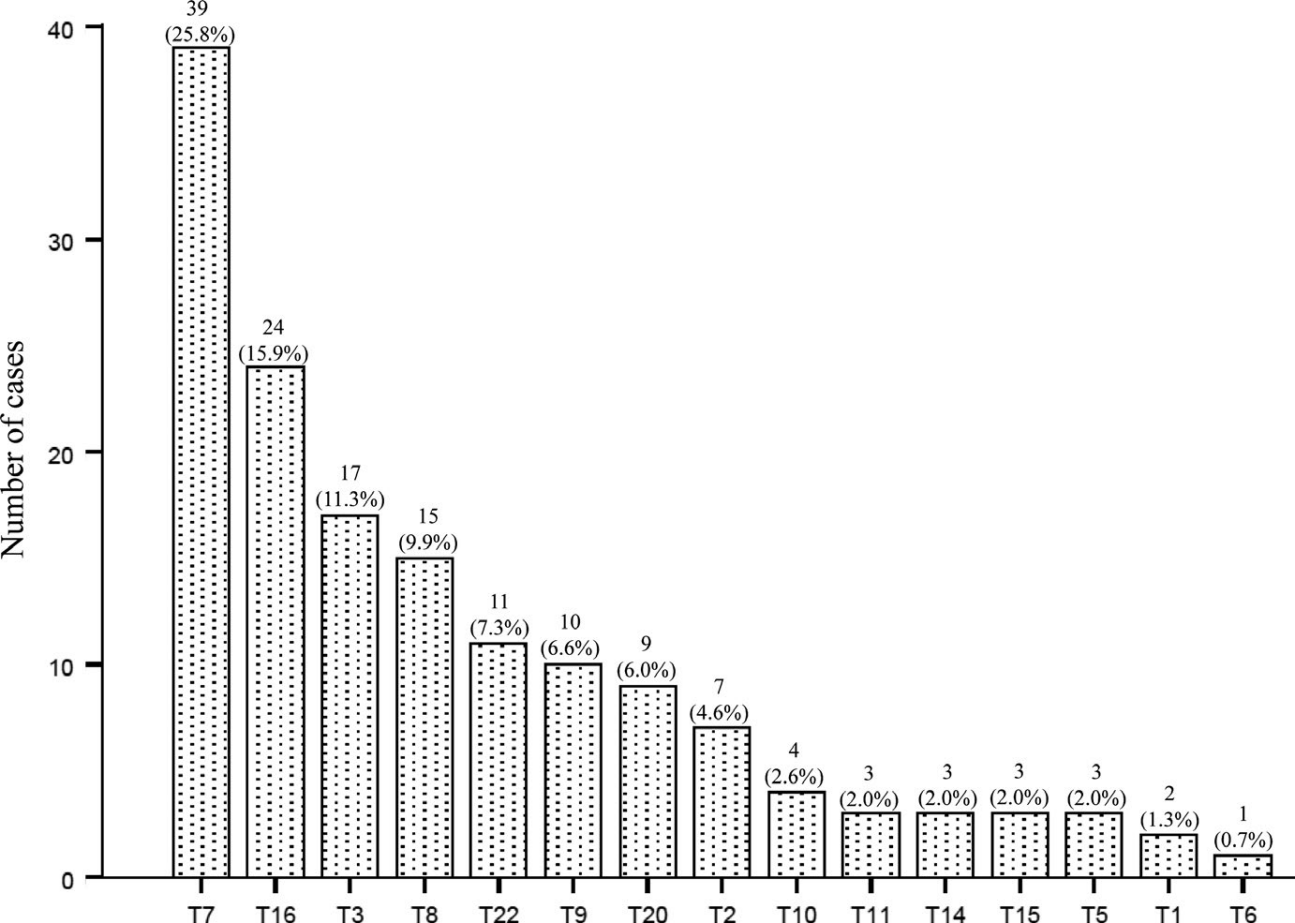
Distribution of rare autosomal trisomies. T, trisomy

We retrospectively reviewed the data of 62,752 NIPS examinations performed in our centre from January 2014 to December 2020. The mean age of these pregnant women was 31.6 ± 4.8 years, and the mean GA was 17.7 ± 2.5 weeks. In total, 151 RATs results were reported (0.24%). Trisomy 7 (T7), T16, T3, T8, T22, T20, T9 and T2 were the most frequently detected RATs, whereas T4, T12, T17, and T19 were not detected (Figure 2).

### 3.2 Confirmation of RATs results

Among the 151 RATs cases, 65 women chose to undergo amniocentesis. The results showed three cases of confirmed true fetal mosaicism (TFM) for RATs, including one case of mosaic T9 and two cases of mosaic T22. Therefore, the positive predictive value of RATs was 4.6% (3/65). We also found six cases of uniparental disomy (UPD), including two cases involving chromosome 2 and four cases involving chromosome 16. There was an incidental finding of one case with confirmed fetal 22q11.2 microduplication. The other 55 (84.6%) women showed a normal karyotype in their amniocentesis results. The detailed data are shown in Table 1.

**TABLE 1:** Clinical outcome data of 65 RATs cases with confirmation

Case No.	Gestation at NIPS (wk)	Age at NIPS (yrs)	Fetal fraction %	NIPS result	Prenatal diagnosis results	Summary of pregnancy outcome
1	12.57	39	11.099	T1	normal	live birth
2	14.86	38	7.711	T2	arr[hg19] 2q24.3(164,883,185–165,471,899) ×4 arr[hg19] 2p14p11.2(68,531,636–87,053,152) ×2 hmz arr[hg19] 2q32.2q37.1(191,219,849–232,206,620) ×2 hmz	UPD, TOP
3	18.00	35	none	T2	arr[hg19] 2p25.3q37.3(12,770–242,782,257) ×2 hmz	UPD, preterm birth
4	15.43	28	3.872	T3	normal	preterm birth
5	22.43	26	9.271	T3	normal	live birth
6	18.86	35	7.931	T3	normal	live birth
7	14.71	27	9.675	T3	normal	live birth, full‐term infants small for gestational age (2100 g)
8	13.71	33	11.73	T3	normal	live birth
9	16.71	31	10.625	T3	normal	live birth
10	13.00	23	4.956	T3	normal	live birth
11	20.43	44	none	T3	normal	live birth
12	16.00	31	none	T3	normal	live birth
13	17.29	30	none	T5	normal	live birth
14	18.71	33	7.623	T7	normal	live birth
15	14.29	31	14.951	T7	normal	live birth
16	13.86	28	7.438	T7	normal	live birth
17	13.86	28	7.438	T7	normal	live birth
18	16.14	33	15.05	T7	normal	live birth
19	18.00	27	7.252	T7	normal	live birth
20	14.29	27	12.527	T7	normal	live birth
21	17.29	32	11.037	T7	normal	TOP for personal reason
22	22.86	35	16.513	T7	normal	live birth
23	17.00	31	13.716	T7	normal	live birth
24	19.57	28	13.098	T7	normal	live birth
25	19.57	32	8.602	T7	normal	live birth
26	19.43	26	11.385	T7	normal	live birth, full‐term infants small for gestational age (2400 g)
27	17.71	28	9.965	T7	normal	live birth
28	16.14	37	10.299	T7	normal	live birth
29	18.71	25	10.775	T7	normal	live birth
30	16.29	38	8.312	T8	normal	live birth
31	19.71	28	10.959	T8	normal	live birth
32	19.71	31	22.302	T8	normal	live birth
33	16.57	26	5.621	T8	normal	live birth
34	18.00	27	none	T8	arr[hg19]9p21.3(25,071,212–25,600,438) ×3	live birth
35	16.14	37	7.873	T9	normal	live birth
36	18.86	26	11.665	T9	normal	live birth
37	18.29	34	5.556	T9	arr[hg19] (9) ×2–3	TFM, TOP
38	15.86	36	4.811	T11	normal	preterm birth
39	15.29	33	4.643	T14	normal	TOP for personal reason
40	15.71	30	6.851	T15	normal	live birth
41	23.00	35	13.305	T16	normal	live birth
42	19.57	31	15.994	T16	normal	single umbilical artery, preterm birth
43	16.71	36	11.058	T16	arr[hg19]16p13.3p13.2(94,807–8,418,576) ×2 hmz	UPD, live birth
44	18.43	26	13.63	T16	arr[hg19]16q22.1q24.3(69,860,932–90,146,366) ×2 hmz	UPD, preterm birth
45	18.00	32	5.505	T16	normal	live birth
46	16.86	40	10.459	T16	normal	preterm birth
47	18.86	32	9.574	T16	normal	miscarriage
48	16.00	26	7.821	T16	normal	preterm birth
49	18.00	37	none	T16	arr[hg19]2p25.3q37.3(12,770–242,782,257) ×2 hmz	UPD, preterm birth
50	17.00	27	none	T16	arr[hg19]16p13.3p13.12(110,582–8,590,518) ×2 hmz	UPD, preterm birth
51	17.57	38	none	T16	normal	abnormal ultrasound, TOP
52	16.14	35	none	T16	normal	live birth
53	12.86	35	8.874	T20	normal	live birth
54	19.57	37	8.255	T20	normal	live birth
55	18.86	32	9.017	T20	normal	live birth
56	12.86	30	15.852	T20	normal	live birth
57	20.14	38	12.836	T20	normal	live birth
58	23.29	34	none	T20	normal	preterm birth
59	23.71	45	none	T20	normal	no outcome data
60	18.71	38	11.172	T22	normal	preterm birth
61	17.43	29	13.028	T22	arr[hg19] 22q11.21(18,919,477–21,464,764) ×3	abnormal ultrasound, TOP
62	18.00	33	18.509	T22	normal	live birth
63	19.00	33	11.295	T22	arr[hg19] (22) ×2–3	TFM, TOP
64	18.86	29	16.377	T22	arr[hg19] (22) ×2–3	TFM, miscarriage
65	15.71	27	none	T22	normal	preterm birth

Abbreviations

GA, gestational age; NIPS, non‐invasive prenatal screening; RAT, rare autosomal trisomy; T, trisomy; TFM, true fetal mosaic; TOP, termination of pregnancy; UPD, uniparental disomy.

### 3.3 Pregnancy outcome results

Among the 151 RATs cases, 147 (97.4%) women were successfully followed up to retrieve the pregnancy outcome. The results showed that eight (5.4%) women personally chose termination of pregnancy without medical indications. Among the other 139 pregnancies, 26 (18.7%) ended with preterm birth, 5 (3.6%) ended with miscarriage and 10 (7.2%) were terminated because of fetal defects including 6 ultrasound abnormalities, 1 confirmed case of fetal UPD and 3 confirmed cases of fetal chromosomal abnormalities. The other 98 (70.5%) pregnancies ended in full‐term live births with normal phenotypes. The detailed information of the RATs cases and their outcomes are shown in Table 1 and Table 2. Birthweight information was available in 107 of the 124 newborns. We found that 13 newborns were recorded with birthweight below 3rd percentile (13/107, 12.1%), including 7 cases of T16, 2 cases of T2 and single case of T3, T15, T20, T22, respectively (Table S1).

**TABLE 2:** Clinical outcomes for 147 cases with RATs results

	TOP directly	UPD	Abnormal ultrasound finding	Fetal abnormal chromosome	Miscarriage	Preterm birth	Full‐term birth	Total
Trisomy 1	0	0	0	0	0	1	1	2
Trisomy 2	0	2^a^	1	0	0	3	2	7
Trisomy 3	1	0	0	0	1	1	14	17
Trisomy 5	0	0	0	0	0	0	2	2
Trisomy 6	0	0	0	0	0	1	0	1
Trisomy 7	1	0	0	0	0	2	35	38
Trisomy 8	1	0	0	0	0	0	14	15
Trisomy 9	1	0	2	1	1	0	5	10
Trisomy 10	0	0	0	0	0	0	4	4
Trisomy 11	0	0	0	0	0	2	1	3
Trisomy 14	1	0	0	0	0	0	2	3
Trisomy 15	0	0	0	0	0	1	1	2
Trisomy 16	1	4^b^	2	0	2	11^c^	8	24
Trisomy 20	0	0	0	0	0	1	7	8
Trisomy 22	2	0	2	3^d^	1	3	2	11
Total	8	6	6	4	4	22	97	147

Abbreviation

RAT, rare autosomal trisomy; TOP, termination of pregnancy; UPD, uniparental disomy.

^a^ Including 1 with preterm birth.

^b^ Including 3 with preterm birth, and 1 with full‐term birth.

^c^ Including 1 with neonatal demise.

^d^ Including 1 with miscarriage, and 1 with abnormal ultrasound findings.

### 3.4 Comparison of pregnancy outcomes between RATs results and low‐risk results

To further investigate the clinical significance of the RATs results, we compared the pregnancy outcomes of women with RATs results obtained by NIPS versus women with low‐risk NIPS results, which were used as the control group (Figure 1 summarizes the study design). We found that the risk of adverse outcomes, including miscarriage, ultrasound abnormalities and preterm birth, was significantly higher in the RATs group than in the control group (*p* < 0.05). We next focused on T7, T16, T3, T8, T22, T9, T20 and T2, which had higher detection rates than the other RATs in our cohort (Figure 2). Compared with the control group, the occurrence of miscarriage was significantly higher in women with T16 results; ultrasound abnormalities were significantly higher in women with T16, T22 and T9 results; and preterm birth was significantly higher in women with T16, T22 and T2 results. However, no significant difference in the occurrence of adverse outcomes was found between the control group and women with T7, T3, T8 and T20 results (Table 3).

**TABLE 3:** Comparison pregnancy outcomes of NIPS results between RATs group and control group

Outcome	Control	RATs		Trisomy 7		Trisomy 16		Trisomy 3		Trisomy 8		Trisomy 22		Trisomy 9		Trisomy 20		Trisomy 2	
	*n* = 10,397	*n* = 147	*p* value	*n* = 38	*p* value	*n* = 24	*p* value	*n* = 17	*p* value	*n* = 15	*p* value	*n* = 11	*p* value	*n* = 10	*p* value	*n* = 8	*p* value	*n* = 7	*p* value
Miscarriage	84	4	0.034*	0	>0.999	2	0.017*	1	0.130	0	>0.999	1	0.086	1	0.079	0	>0.999	0	>0.999
Abnormal ultrasound finding	82	6	0.001*	0	>0.999	2	0.016*	0	>0.999	0	>0.999	2	0.003*	2	<0.001*	0	>0.999	1	0.055
Preterm birth	365	26	<0.001*	2	0.388	11	<0.001*	1	0.456	0	>0.999	3	0.006*	0	>0.999	1	0.249	3	0.001*

Note

NIPS, non‐invasive prenatal screening; RAT, rare autosomal trisomy. *p* values comparing pregnancy outcomes of NIPS between RATs group and control group are obtained from χ^2^ test or Fisher's exact test. *p < 0.05

## 4 DISCUSSION

The potential clinical implications of detecting fetal RATs during prenatal testing are unclear, and most of these results to date have been obtained from chorionic villus sample (CVS) analysis. With the rapidly increasing application of NIPS, more RATs results are being obtained by this method; however, adequate outcome data with which to determine the clinical implication of these RATs results are still lacking. Because both NIPS and CVS analysis involve analyzing the genetic component of the placental trophoblastic cell lineage, NIPS results are considered comparable to CVS short‐term culture results.^11^ However, several differences still exist between CVS analysis and NIPS. Van Opstal et al.^12^ reported that NIPS is more sensitive to (low‐level) placental mosaicism involving the cytotrophoblast than in CVS analysis. Additionally, Benn et al.^12^ showed that there was a significantly higher frequency of TFM among RATs ascertained by NIPS (9.8%) than CVS analysis (3.0%). These differences may result in different conclusions regarding the RATs results obtained by CVS analysis versus NIPS.^13^ Therefore, it is beneficial to clarify the clinical implications of RATs detected by NIPS.

The detection rate of RATs by NIPS reportedly ranges from 0.12% to 1.03%,^13^ and almost all RATs also exhibit mosaicism.^8^ However, there is no consensus regarding the association between RATs detected by NIPS and adverse pregnancy outcomes according to recent reports.^3^, ^5^, ^8^, ^14^ Pertile et al.^5^ reported that the presence of a RAT found by NIPS was associated with an increased risk of feto‐placental disease, including miscarriage, intrauterine fetal death, intrauterine growth restriction, TFM and UPD, and only 27% (14/52) of RATs with outcome data resulted in a normal live birth in their study. Scott et al.^14^ found that 16 (57.1%) of 28 cases of RATs had abnormal outcomes, including 6 miscarriages, 5 TFMs and 5 fetal structural anomalies on ultrasound. However, He et al.^8^ found no sonographic structural anomalies or TFM among 61 cases of RATs, and 54 cases were confirmed to have confined placental mosaicism. Pregnancy follow‐up showed that 95% of the women in their study had an uncomplicated pregnancy with the exception of two cases of intrauterine growth restriction (one case of T16 and one case of compound T7/8).^8^ In our study, 29.5% of RATs cases were associated with preterm birth, miscarriage, ultrasound abnormalities, TFM and UPD, and the other 70.5% of pregnancies ended in normal full‐term live births. These differences in RATs outcomes may be associated with the distribution of the GA at the time of NIPS. In the studies by Pertile et al.^5^ and Scott et al.,^13^ most samples were collected in the first trimester, which was earlier than in the study by He et al.^8^ and the present study. On the other hand, a nuchal translucency scan or a second‐trimester ultrasound before blood sampling was mandatory in the study by He et al.,^8^ which further reduced the number of high‐risk pregnancies compared with other studies. Finally, the small sample sizes in every study may have also contributed to the differences.

T16 is the most frequently reported trisomy and might be associated with a risk of adverse pregnancy outcomes.^11^, ^15^, ^16^ Benn et al.^13^ reviewed six studies and found that 64.5% of pregnancies with T16 had fetal abnormalities, fetal growth restriction and miscarriage. Grati et al.^11^ extrapolated CVS data to NIPS and considered that only T16‐confined placental mosaicism had a strong association with an increased incidence of birthweight below the third percentile and preterm delivery, whereas other RATs were associated with a low incidence of adverse outcomes. Recently, Peng et al.^17^ found 5 true‐positive results and 9 false‐positive results among 14 cases of T16 detected by NIPS. Of the nine false‐positive cases, eight infants were born with a low birthweight. The authors also found two premature infants and considered that T16 pregnancies might be at higher risk for preterm delivery. In our study, a significant association was found between T16 and preterm delivery, miscarriage, and ultrasound abnormalities as compared with normal controls. Similar with previous reports, we also found T16 newborns have an increased incidence of low birthweight (43.8%, 7/16). According to the above results, genetic counselling and pregnancy management should be implemented when NIPS reveals T16, especially for preterm delivery.

Like T16, T22 is also easily detectable and is potentially associated with adverse outcomes.^5^, ^7^, ^13^ Benn et al.^13^ identified 17 cases of T22 with outcome information available from recent reports, of which 10 experienced fetal loss, 2 were diagnosed with TFM, 1 was diagnosed with fetal growth restriction and 4 had an apparently normal outcome. Pescia et al.^7^ reported that 100% (3/3) of T22 results were confirmed to be associated with TFM among 19 cases of RATs detected by NIPS. Other studies have shown that fetuses with mosaicism for T22 at the time of amniocentesis can be associated with abnormal ultrasound findings.^3^, ^18^, ^19^ In our study, 33.3% (2/6) of T22 results were confirmed to be associated with TFM of T22, and T22 was associated with both ultrasound abnormalities and preterm delivery. Therefore, ultrasound and invasive prenatal diagnosis should be recommended for women with T22 results.

T7 is the most common RAT, with a rate of 0.056% among NIPS examinations, and has a mostly favourable outcome.^20^ Zhu et al.^20^ reviewed 85 cases of T7 detected by NIPS. Among them, 88.2% were live births with a normal outlook; 14.1% had intrauterine growth restriction, preterm birth or low birth weight; 3.5% presented with ultrasound abnormalities; and no fetal loss was observed. He et al.^8^ analyzed 24 cases of T7 and reported that all had normal pregnancy outcomes, and T7 was not confirmed in any cases. Our results also showed no significant difference between the T7 group and control group. Additionally, like T7, the pregnancy outcomes of T3, T8 and T20 were generally satisfactory in our study. Therefore, caution is needed before performing any invasive procedures in pregnancies with these RATs, which may be associated with a favourable outcome.

The main strength of this study is the relatively large number of RATs results with pregnancy outcomes from a single tertiary centre. We systematically and comprehensively expounded the relationship between various RATs detected by NIPS and adverse pregnancy outcomes for the first time, and our data showed that the associations and relevance for each chromosome were different. The results could be informative in clinical counselling and pregnancy management.

## 5 CONCLUSIONS

In this study, we retrospectively analyzed 151 RATs detected by NIPS and found that the chances of adverse outcomes, including miscarriage, ultrasound abnormalities and preterm birth, were significantly higher in the RATs group than the control group. Among the most frequent RATs, T16, T22, T9 and T2 were associated with adverse outcomes, and more intensive pregnancy monitoring should be provided for these special cases. Given the low positive predictive value and various outcomes, the question regarding whether it is beneficial to extend NIPS for RATs remains unanswered. More clinical data are needed to further evaluate the clinical implications of these RATs results.

## CONFLICT OF INTEREST

The authors confirm that there are no conflicts of interests.

## AUTHOR CONTRIBUTIONs

**Ying Lin:** Data curation (equal); Formal analysis (lead); Writing – original draft (equal); Writing – review & editing (equal). **Ping Hu:** Formal analysis (equal); Investigation (equal). **Hang Li:** Data curation (equal). **Chunyu Luo:** Investigation (equal). **Dong Liang:** Funding acquisition (equal); Project administration (equal); Writing – review & editing (equal). **Zhengfeng Xu:** Investigation (equal); Project administration (equal).

## Supporting information

Table S1

## Data Availability

The data described in this study are available upon request from the corresponding authors.
