# Supplementary material for: Pregnancy outcomes of rare autosomal trisomies results in non‐invasive prenatal screening: clinical follow‐up data from a single tertiary centre
Source: J Cell Mol Med. 2022 Feb 16;26(8):2251–8. doi: 10.1111/jcmm.17245 (PMC8995450; doi:10.1111/jcmm.17245)
Supplement: Supplementary file 1 — Table S1 [file JCMM-26-2251-s001.docx]

|  | birthweight <3rd percentile  (n) | Total  (n) |
| --- | --- | --- |
| Trisomy 1 | 0 | 2 |
| Trisomy 2 | 2 | 5 |
| Trisomy 3 | 1 | 12 |
| Trisomy 5 | 0 | 2 |
| Trisomy 7 | 0 | 37 |
| Trisomy 8 | 0 | 11 |
| Trisomy 9 | 0 | 4 |
| Trisomy 10 | 0 | 3 |
| Trisomy 11 | 0 | 2 |
| Trisomy 14 | 0 | 2 |
| Trisomy 15 | 1 | 1 |
| Trisomy 16 | 7 | 16 |
| Trisomy 20 | 1 | 6 |
| Trisomy 22 | 1 | 4 |
| Total | 13 | 107 |

Table S1 Distribution of cases with birthweight <3rd percentile in 107 newborns.
